# DLT Based Authentication Framework for Industrial IoT Devices

**DOI:** 10.3390/s20092621

**Published:** 2020-05-04

**Authors:** Cristian Lupascu, Alexandru Lupascu, Ion Bica

**Affiliations:** Faculty of Information Systems and Cyber Security, “Ferdinand I” Military Technical Academy, 050141 Bucharest, Romania; cristian.lupascu@mta.ro (C.L.); alexandru.lupascu@rcpu.eu (A.L.)

**Keywords:** distributed ledger technology, industrial Internet of Things, decentralized authentication, industry 4.0, secure multi-party computation

## Abstract

The latest technological progress in the industrial sector has led to a paradigm shift in manufacturing efficiency and operational cost reduction. More often than not, this cost reduction comes at the price of dismissing information security, especially when multiple stakeholders are involved and the complexity increases. As a further matter, most of the legacy systems and smart factoring processes lack a security by design approach, making them highly vulnerable to cyber-attacks. Taking into consideration the aforementioned issues, we propose an architectural framework for Industrial Internet of Things (IIoT) that provides authentication and guaranteed integrity. Our proposal properly addresses the security by design principle while combining some of the emerging technologies like Secure Multi-Party Computation (SMPC) for grounded policy rules and Distributed Ledger Technology (DLT) for an immutable and transparent registry.

## 1. Introduction

The industry has gone through different stages while being reshaped by four big industrial revolutions. It all began in the 18th century when the first revolution (Industry 1.0) was started by the weaving loom, more exactly in 1784. After the development of steam and water-powered machines, the next industrial revolution (Industry 2.0) started around the 20th century with machines powered by electrical energy. The major advances in the electronics industry over the last years of 20th century brought the third revolution (Industry 3.0) with a landmark invention named Programmable Logic Controller (PLC). The first one was built in the 1960s and represented a groundbreaking success for the automation of manufacturing systems.

Last, but not least, the fourth industrial revolution (Industry 4.0) emerged together with the evolution of Internet and communications. The paradigm has changed from the traditional view of manufacturing to a new perspective where the boundaries of the physical world overlap with the virtual world. This boundary is very blurry and includes a large range of technologies as: Cyber Physical System (CPS), IIoT, cloud, edge (fog), and cognitive computing.

The main idea around Industry 4.0 is the integration of those worlds, shaped by a physical–digital–physical flow of information which occurs continuously through three simple steps: physical to digital, digital to digital, and digital to physical. The first step is to capture the data from IIoT and other sensors in order to create a digital twin. The second step consists in processing all the digital records in the process which can be enriched using cutting edge technologies. Those enrichments could be any of the following: finding optimizations by using High Performance Computing (HPC), perform analytics and create insights of the process, automate and decentralize decisions using artificial intelligence and distributed consensus. The last step consists of transmitting the decisions to the physical world as a feedback circuit.

### 1.1. Motivation

Edge devices are linked together in a fog network where all the data is collected. Given the fact that IIoT sensors were designed to operate in limited conditions where computing power and battery resources are scarce, these sensors tend to have some security drawbacks. Out of necessity, the industry developed smarter sensors, capable of more complex operations. However, there is still a gap between the required security controls and the ones provided by the lightweight designed architectures. Very few implementations have a security by design approach and most of the devices in use are very old with unaddressed issues.

Another rising issue is the authenticity of an IIoT sensor, either if we are talking about the counterfeited devices or if we are talking about malicious updates on the firmware. The lack of support in cryptographic primitives impacts the use of traditional schemes for authentication, identification and integrity assurance in the sense that they are not well suited for this type of device, thus emphasizing the necessity of new approaches for information security. Distributed ledger technologies bring to the table an open registry that can help us address the aforementioned issue by taking decentralized decisions based on consensus.

Current IIoT devices are developed with a strong motivation to keep it as lightweight as possible and to reduce any unnecessary functionalities due to cost reasons. This approach steered the implementation to a vulnerable position against the latest cyber-attacks. Lately, some responsible organizations within the European Union, namely European Union Agency for Cybersecurity (ENISA) started to issue security analysis reports, recommendations and good practices for current challenges, but with all the effort there is a security gap between the upcoming devices and the outdated ones already present in many smart manufacturing factories.

### 1.2. Current Issues

In the following section, we go through some examples of what could go wrong with the IIoT sensors and, more important, what we can learn from those mistakes. The first and probably the most notable one is Stuxnet. This threat targeted Supervisory control and data acquisition (SCADA) systems which were running Siemens SIMATIC® WinCC or SIMATIC® Siemens STEP 7 software, used in both the overview process and in the control system of a nuclear power plant. The goal of this cyber-attack was to sabotage the PLCs responsible for the centrifuges and to reprogram them in order to physically destroy them. They successfully managed to cause substantial damage to Iran’s uranium enrichment process. Skipping the details about the features and how the infection propagated, it was a dormant worm that was active somewhere around five years until discovered. The infection lifespan could have been much shorter if some alerts had been triggered when the behavior of the PLCs changed or when the firmware of those devices was altered.

Stuxnet is just the first from a five known Industrial Control System (ICS) tailored malware developed during the past decade. There was a steady increase in events until 2017, naming here the BlackEnergy2 in 2010 and Havex in 2013, the first one gaining notoriety in 2008 when it was reported to have been used in the cyber-attacks against Georgia and the latter being a part of a widespread espionage campaign across numerous industries like energy, aviation, pharmaceutical, defense, and petrochemical. In 2017, together with the EternalBlue vulnerability and the subsequent ransomware like WannaCry or NotPetya, we witnessed a dramatic expansion in ICS security shortage highlighted by two events: the cyber-attack on Ukraine’s powers grid on 17 December 2016 which cut off 20% of Kiev’s power for one hour and the discovery of compromised safety systems in a Saudi Arabian petrochemical plant.

The Industroyer, also known as Crashoverride, is responsible for the cyber-attack on Ukraine’s electrical grid and is the first ever known malware specifically designed to attack power grids. In the case of the Saudi Arabian petrochemical plant, a unique attack compared to other ICS attack attempts, named Triton or Trisis, targeted safety instrument systems responsible for ensuring a safe fail state even in case of a malfunction.

There are certainly even more types of attacks on Internet of Things (IoT) systems, for example in the healthcare sector more than 465,000 patients had implantable cardiac pacemakers from Abbott (formerly called St. Jude Medical) vulnerable to hacking [1]. In the automotive industry, with the Jeep Hack [2] back in 2015, where authors used the vehicles Controller Area Network (CAN) bus to exploit a firmware update vulnerability. Another interesting type of attack is the Brickerbot botnet or the Silex malware which tries to brick IoT devices by deleting the storage, iptables rules, and network configuration and eventually halts the device. Counterfeiting is yet another rising problem, especially when the General Data Protection Regulation (GDPR) [3] makes organizations liable if counterfeits are unwillingly accessing clients data, the best example here is the big hack into Super Micro [4].

All of these attacks have one thing in common—the lack of security capabilities in lightweight IoT sensors which is augmented with the lack of emphasis from the device manufactures that do not take it seriously and do not properly perform penetration tests on their smart sensors. Also, out in the wild, there are still vulnerable devices that have zero protection, by design or by other means, even against known malware, not to mention zero day flaws.

### 1.3. Research Contribution

The novelty of the paper consists in a framework that can authenticate devices by learning their behavior and limits of measured values. All the process is transparent thanks to a distributed registry which also assembles a decentralized decision if a device is corrupted or not. The decisions are taken into consideration based on policies that are distributed through different Secure Multi-Party Computation (SMPC) nodes to ensure that the framework is properly working even if some parties are being dishonest.

In the next section, the background of the technology we used in the proposed solution together with relevant related work will be presented. In Section 3, we describe our main contribution, which is a Distributed Ledger Technology (DLT) based authentication (and integrity assurance) framework which can detect when specific sensors are changed, in terms of their recorded values or when their behavior is deviated from the rest. We present the experimental results in Section 4 and conclude the paper in Section 5.

## 2. Background and Related Work

### 2.1. Overview of Distributed Ledger Technology

Distributed ledger technology, also popularly known as blockchain technology, has its roots in a paper written by an unknown group of people under the pseudonym Satoshi Nakamoto [5]. This paper highlights a blueprint of a decentralized digital currency which can be exchanged between users over the Internet using a peer-to-peer network. The exchange of this digital currency, or any other digital asset, is achieved by issuing a transaction signed by the user initiating the transfer. Furthermore, these transactions are packed into blocks using a Merkle tree structure [6,7], while blocks are chained together by including in each one the hash of the previous block. These decentralized transactions can be verified by the nodes, which are connected to each other in a peer-to-peer network and relay new information by gossip. Each node maintains a copy of the distributed ledger represented by the ordered sequence of blocks that make up the ‘world state’ at a specific point in time. In other words, the ‘world state’ can be viewed as a state machine.

A distributed ledger can only be modified by issuing a transaction to one of the peers who will broadcast it to the blockchain network. Each node, or a subset of validator nodes, will perform basic validation of this pending transaction, for example by checking the signatures and the format. After validation, each pending transaction will be put in the local transaction pool, from where transactions will be fetched to build a potential next block. As nodes will have different next block candidates, they run a consensus mechanism to decide who is allowed to propose the next block, thus ensuring a clear order of transactions. When a new block is broadcasted into the blockchain network, each node performs another validation of each transaction within that particular block by also using the state machine information to ensure consistency.

### 2.2. Overview of Secure Multi-Party Computation

Secure multi-party computation represents a subarea in cryptography that has an ultimate goal to enable multiple parties to jointly compute a function (or a circuit) over their input data while keeping the privacy. Given *n* participants, each one with his private data:

(1) $$(p_{1},p_{2},\ldots ,p_{n})\;\mathrm{participants}\;\mathrm{and}\;\left(d_{1},d_{2},\ldots d_{n}\right)\;\mathrm{data}\;\mathrm{sets}$$

could compute the value of a function from private data sets:

(2) $$F\;(d_{1},d_{2},\ldots ,d_{n})=\mathrm{SMPC}\;\mathrm{Protocol}\;(p_{1},p_{2},\ldots ,p_{n},d_{1},d_{2},\ldots ,d_{n})$$

while keeping the inputs secret.

The first formal introduction was in 1982 by Yao with the Millionaires’ Problem [8], which represents a particularity of a two-party computation. In the following years, the two-party case was completed with a generalization [9] by Goldreich, Micali, and Wigdserson, a model in which the computation is based on secret sharing of all the private inputs and the use of zero-knowledge proofs (ZKP) to protect against a malicious case. The previously mentioned work had set a general basis for all future multi-party protocols for secure computations.

Many of the current implementations of two-party SMPC schemes are based on the oblivious transfer, a protocol first introduced by Rabin [10] that allowed to send a message with 50% probability while the sender remains oblivious whether the receiver got the message or not. After this paper had been published, other useful forms have been developed: one out of two oblivious transfer [11] and the generalized *k* out of *n* oblivious transfer [12] where one could extract an information from a database without the server getting to know which information was retrieved, and without allowing the user to know anything else about the other elements not included in query. All the oblivious transfer protocols represent an enhanced version of the private information retrieval (PIR) protocols introduced in 1995 [13].

Generally speaking, the SMPC has two main categories: two-party and multi-party computations. The former is briefly described above while the latter is further classified based on the underlying sharing secret scheme: Shamir secret sharing [14] and additive secret sharing. Regarding the threat model of these schemes, there are usually two types of adversaries: semi-honest (passive) ones—curious parties that can cooperate to gather information about the protocol but they respect the protocol specifications and the malicious (active) ones that may deviate from the protocol execution in order to cheat.

Shamir secret sharing schemes can tolerate up to *t* adversaries from a total of *n* parties while achieving information-theoretic security with respect to:

(3) $$t<\frac{n}{3}$$

in an active (malicious) setup and:

(4) $$t<\frac{n}{2}$$

when we have a passive setup.

Additive secret sharing schemes can be tolerant to:

(5) t<n

adversaries, both in the passive and active setup with the adversary having unbounded computational power.

In the proposed solution, we chose the two-party implementation of an open source library, as our main goal is to remove centralized high value targets and converge towards a decentralized approach. Also, the preferred underlying secret sharing scheme is Shamir because is more suitable than additive secret sharing schemes to complex circuits like distributed signing operations. The SMPC component could be easily swapped with a multi-party implementation for a production environment.

### 2.3. Related Work

There are related papers in this field proposing frameworks based on the distributed ledger technologies for the IoT world. In their work [15], Shahid Raza et al. made a survey on the existing blockchain IoT solutions: IOTA [16] which is a permission-less cryptocurrency to enable technological resource trade, another interesting proposal is the Guardtime KSI [17], a keyless signing infrastructure which provides data integrity through the use of hash-based digital signatures (similar to IOTA). X-Road is the backbone of Estonia’s e-government services and it is secured by the KSI blockchain. Another analysis of the challenges between blockchain and IoT can be found in [18].

IBM’s solution is a private blockchain (e.g., Hyperledger Fabric [19]) and uses Practical Byzantine Fault Tolerance (PBFT) as a consensus protocol, enabling the network to continue working properly even if only one-third of nodes are honest. The cryptographic primitives are based on asymmetric encryption which may not be a suitable solution for the limited IoT sensors, but rather for more resourceful devices (e.g., proxies).

Another DLT approach is the Enigma [20], a decentralized computation platform that addresses privacy guarantees. The paper is focused on the privacy of the collected data, ensured by a secure multi-party computation protocol. Information is stored on a distributed hash table (DTH), but sensitive information is encrypted in an off-chain database and the references together with the policies are stored on-chain, following that SMPC nodes process encrypted data. At the moment, the solution is implemented with Intel Software Guard Extensions (SGX) and the SMPC approach is closely on the roadmap.

In paper [21], authors proposed a consumer-oriented IoT lightweight authorization stack for smart-home applications, where devices connected to the cloud exchange authorization input commands to a user’s smartphone. With a focus on the device-user link, the architecture addresses security issues in an untrusted cloud environment.

FlexSMC represents a management and orchestration framework for secure multi-party computations described in [22], which addresses the issue of dynamic environments such as IoT networks mixed with privacy-preserving collaborative computations. This approach focuses on the virtual centrality of SMPC nodes, serving as a single point of data contact for requests, and eventually translates them into SMPC sessions. If there is a need to address the management and monitoring of IoT devices one can choose to use the improved architecture presented in [23] using a private blockchain. 

Smart buildings and smart home IoT infrastructures are analyzed in [24] in order to produce an IoT security framework, along with a threat model that is useful when developing a security protection methodology against cyber-attacks. The paper also shows that intrusion detection systems based on anomaly behavior analysis (ABA-IDS) can successfully detect a wide range of attacks on IoT devices. Another extensive analysis of the challenges, privacy and security needs of smart home environments can be found in [25] that pinpoints various threats and solutions.

Several European Union (EU) research projects such as European Union Horizon 2020 Research and Innovation project Safe-Guarding Home IoT Environments with Personalised Real-Time Risk Control (GHOST [26]) aim to develop a safe IoT environment by raising the effectiveness of automation in existing cyber security services by using blockchain. SecureIoT, a major industry oriented EU project focuses on security data collection, monitoring, and predictive analytics mechanisms. Its main concept relies on foretelling and anticipation of security behavior of IoT entities [27,28].

We must mention that this is not the first DLT based authentication framework, for example Gaurav et al. proposed an authentication protocol for cloud database using blockchain [29]. The novel mechanism is formally tested against denial of service, impersonation, offline guessing, and reply attacks, showing that the methodology is secure.

Another DLT based platform to assure data integrity can be found at [30], where authors propose a proof of concept implementation based on realistic IoT scenarios using Raspberry Pi devices and Hyperledger Fabric as a permissioned network.

Physical Unclonable Functions (PUFs) on devices represents a low cost primitive exploiting some unique random pattern which could easily be used in key generation and key management. Such a method is proposed in [31] to develop an authentication protocol for IoT. This is certainly not the only PUF based implementation. We find in [32] an authentication alternative with PUF-derived IoT identity in a zero-knowledge protocol [33] which eliminates the costly process of external key generation. Moreover, a new error-resilient programming language that relies on Event-Condition-Action (ECA) rules can be found in [34].

Most of the distributed ledgers deployed in IoT environments make use of cryptographic keys which can become the target of post-quantum attacks. In literature this problem has already been addressed. In a figure shown in [35], authors propose a quantum-secure distributed ledger with a novel one-time signature (OTS) namely, DL-OTS, which offers 75% reduction in signature size and 76% in signature creation times over Winternitz-OTS+ scheme.

The connection between blockchain and IoT for Industry 4.0 is further analyzed in many papers to outline the advantages, recommendations and various challenges when integrating blockchain with IoT (see [36,37]).

Compared with other related implementations, our proposed framework comes with a decentralized authentication process based on consensus, thus avoiding targeted cyber-attacks on infrastructure components. Moreover, the policies which govern the settlements on the DLT are anchored in a multi-party scheme using multiple SMPC nodes that can be seen as master nodes within the blockchain network.

## 3. Proposed DLT Based Authentication Framework

Standardization and certification schemes represent the future for a more secure Internet of Things and the European Telecommunications Standards Institute (ETSI) Technical Committee on Cybersecurity (TC CYBER) made the first step in this direction by releasing the ETSI TS 103 645 [38] standard for cybersecurity in the IoT. The security baseline of this standard brings provisions for the consumer part, however these good practices are as well applicable to the Industrial Internet of Things.

A more focused IIoT standardization effort is made by the Object Management Group (OMG), their works include various standards such as Data Distribution Service [39] (DDS) protocol for network interoperability of connected machines, a dependability assurance framework for safety-sensitive consumer devices or the unified component model for distributed, and real-time and embedded systems.

In this paper, we propose a decentralized framework for the authenticity and integrity checking for the Industrial IoT sensors. More precisely, our goal is to address the following issues in the existing implementations:

- Decisions to exclude malicious devices must not be centralized but rather in a decentralized manner, defined by consensus to avoid targeted cyber-attacks;
- The deciding factor must be very well secured in such a way that if at least half of parties are fair (in a passive setup) then all the commitments are still based on the initially established policies;
- Authentication of devices should not be based on Open Systems Interconnection (OSI) Layer 2/Layer 3 addresses but rather on the behavior (Layer 7);
- Such a framework must not consist in a black box and a centralized database of events but rather in a transparent registry with immutability property.

In this way, we chose to use a distributed ledger for the openness of the infrastructure, where all the information and events could be audited anytime, and more importantly, to be unchangeable without consensus.

### 3.1. Distributed Ledger Component

In the case of DLT, we used a Byzantine-fault tolerant state machine replication. More specifically we used Tendermint [40] to deploy a consensus mechanism over multiple machines performing checks of the measured values from sensors using some predefined thresholds.

Other distributed ledgers instances do exist, however, we will only mention IBM’s Hyperledger Fabric which is a private blockchain framework implementation with a modular architecture. Also, we think that a public DLT, either permissioned or non-permissioned, is not suitable because of speed issues, large unused data overhead, availability, and scalability challenges. 

Block generation time must be extremely short in order to process all the information from the sensors. The effectiveness of a scaled network of sensors can be adjusted from the number of blockchain nodes and SMPC parties. Block generation time plays an important role, especially in real time scenarios where measured values must be immediately acknowledged in order to generate other actions or events. This should take place almost instantly in real time use cases or fast enough to include all the transactions (containing measured data) issued in the latest sampling window (e.g., IIoT sensors sampling new values every minute are confirmed on DLT in one minute maximum), otherwise the information will be posted with delay resulting in a non-synced system. Block generation time is directly dependent by the number of included transactions and the consensus of DLT network.

The importance here of the DLT is two-fold, the main benefit is the security of a distributed architecture where a decision is taken based on consensus, and the second advantage will be the transparency necessary for the external actions that must be taken when some values exceeds the default range.

To verify the trustworthiness of some sensors one should naively approach a method in which their values are stored into the database, then a software component takes them for checks and decides whether that data is valid or corrupted. The events along the history demonstrated that this is not enough in the face of modern cyber-attacks, where the software can be exploited to perform other checks or the values stored in the database can be forged for compliance.

Our proposed technique takes advantage of above mentioned benefits by deploying a number of DLT nodes, where each node has an associated engine that implements the consensus. This consensus consists in validation over the recorded values from sensors against the thresholds within policies, which will also be validated using a public key into the genesis block. Going forward with such an approach it removes all the trusted third parties responsible with the authentication and integrity assurance, and in exchange, it promotes a decentralized manner of taking the decision whether the authenticity and integrity have been violated or not.

**Figure 1:**
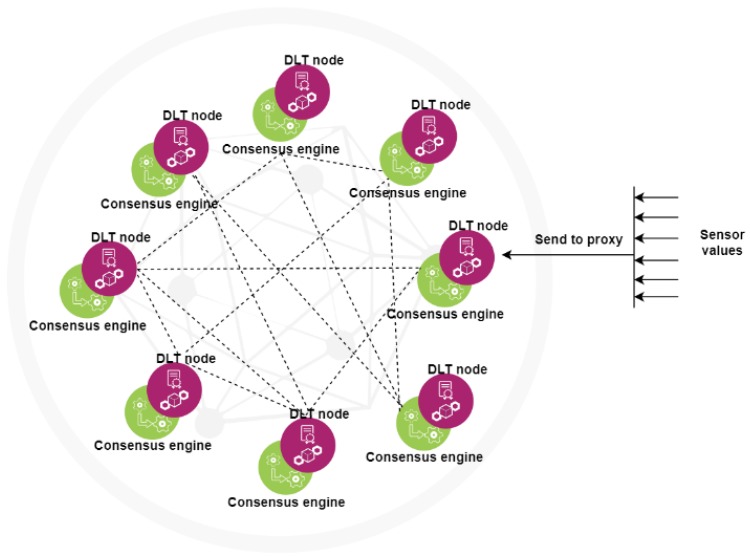
Nodes with the associated consensus engine.

The blockchain nodes and their associated engines can be seen together as a compact edge service running close to sensors (acting like a proxy) while also playing the role of a distributed decision maker. A macroscopic view of this distribution can be seen in Figure 1.

Informational flow occurs from the values sampled by sensors. These values are then recorded in blockchain through a proxy (the closest DLT node) and afterwards the consensus validates them (against the policy).

### 3.2. Secure Multi-Party Computation Component

We implemented the proof of concept for this architecture using the existing SMPC implementation of Unbound Tech solution [41] for crypto asset protection which has an open source 2-party implementation. From this tool, we used the Elliptic Curve Digital Signature Algorithm (ECDSA) operation to sign, using secp256k1 key and the thresholds of sensors values. Of course, there are also other ways to implement this architecture, for example the SMPC implementations of KU Leuven, which is an open source library named SCALE-MAMBA [42]; MPyC [43]—a secure multiparty computation software implemented in python or the licensed implementations like Sharemind MPC [44] which is based on additive secret sharing. The reason for choosing the Unbound Tech open source solution instead of other open source libraries is that it had already implemented the ECDSA circuit over the SMPC primitives, although the implementation of a signing algorithm over SMPC is not a scope of this paper.

The root of trust for this approach will be the policies according to which the consensus will be running on the DLT. The policies will contain the specific thresholds for each type of sensors (heat, humidity, rotation/speed sensors, etc.) and also the hash of each type of firmware which resides on sensors. Since every decision (e.g., if a sensor measures values out of expected range) is based on these values we must assure that no changes appear during this process. 

Digital signing of these policies would be a very efficient way to protect from such an unauthorized alteration, but on the other hand if we choose to implement it on a single party then we will have an increased risk for that specific machine to be compromised (and all the DLT nodes would agree on a wrong consensus). We minimize the risk by splitting the private key which will sign the agreed policies on multiple parties, so the overall chance to compromise the system will be the one in Equation (4) in case of a malicious setup.

Using the open source framework [41] mentioned above, we implemented an ECDSA signature using the secp256k1 key on the Secure Hash Algorithm (SHA256) digest of the policy, which has a JavaScript Object Notation (JSON) format like the following:

{“sensorType”: {“min”:“10”;“max”:“1000”;“firmware_hash”:“71df3b6e89726e61b979d503db929ac225b8494cb33d1 6f792deb56886bdbd76”}}(6)

**Figure 2:**
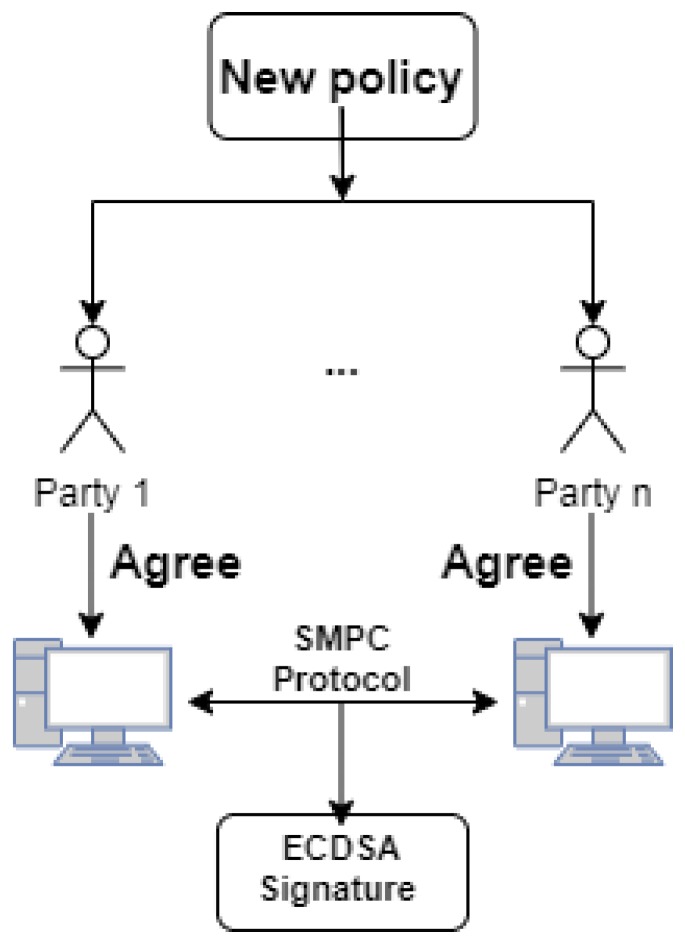
Multi-party computation signing flow of policies.

The inputs for the SMPC component consist in the threshold values along with the hash of firmware, while the output represents the signature, as can be observed in Figure 2.

Role distribution is a major aspect when we want to assure a strong identity and access management, this is why in the above scenario each party should have at least two roles: the administrator of the system which gives access to the application and the second role as the key share owner which will participate in the ECDSA distributed signature operation. For simplicity, in our experiments both roles are joined.

We stress that the role of key share owners is vital for the initial policies setup or further modifications of policies. Without a key share, the signing process fails, therefore we propose the use of an extra layer of role distribution based on any k-out-of-n secret sharing scheme. Each SMPC key share would be encrypted employing a secret (shared to n entities) so that any key share owner could be replaced by n-1 key owners. In a manufacturing plant we can map these roles mainly on machine operators and allow engineers to keep at least half of the SMPC key share owners for system administrators (see Equation (4)).

### 3.3. Overall Framework

The information is collected from sensors in a proxy architecture, meaning that each IIoT device will communicate with the assigned proxy which will sign the information using the wallet private key, and then pass it further to entry/exit point. Each proxy acts as a light node of the blockchain because it will only index the information related to the sensors in his proximity, for reasons of resource optimization. 

The gateway that receives the transactions (signed values of sensors or firmware/file system hash) is a Secure Multi Party Computation node that will apply the policy based on rules that are valid for all the parties which set up the guidelines. This validation process includes the sending of the transaction to the distributed ledger and the waiting delay for the confirmation performed after achieving consensus.

The consensus of the distributed ledger is a process where each node must agree that the values recorded by sensors are pursuant to the values present in the policy. Otherwise, the transaction will be rejected then will save the invalid transaction as plain data to the DLT by publishing a signed message with this information. In case the initial transaction is compliant, the gateway together with the rest of SMPC nodes will sign and post the validation.

The gateway is a special type of distributed ledger node and SMPC party which could potentially employ a linear regression algorithm that detects when the behavior of sensors is changed, taking into account the thresholds from the policies. We must stress out that linear regression algorithms are suggested for a future work and they were not implemented during the experiments. The SMPC nodes can be viewed as master-nodes, however, their essential role in this architecture is to lock the policies and block any unauthorized change unless all managing entities agree.

Our proposed architecture has some prerequisites and considerations regarding the proxy servers and the gateway—namely, because these devices are not lightweight we suppose that they have serious protections against modern cyber-attacks.

In order to operate the proposed framework, there is a low-medium level of expertise required, which is limited to the use of a software application for establishing policies, network configurations so that components could communicate between them, the deployment of DLT nodes on each proxy (and on other resources for a stronger security). However, a misuse of configuration parameters in the distributed ledger could hinder the performance of real time scenarios—as well as its security (depending on the number of nodes).

**Figure 3:**
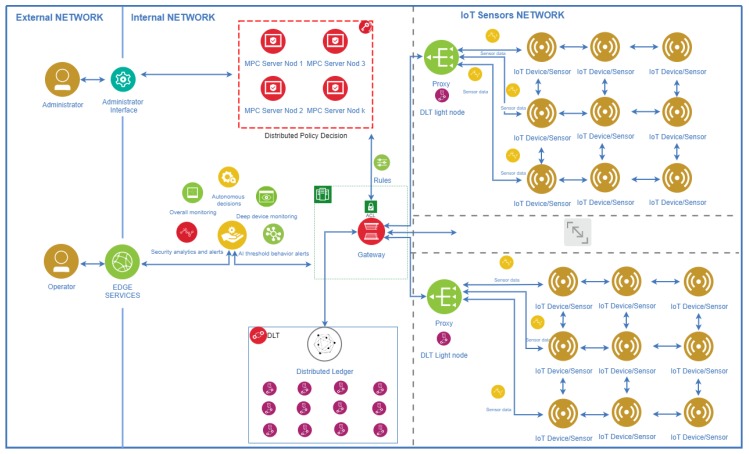
The authentication framework.

Firmware integrity can be achieved by anchoring the firmware hash into the policy, then read the firmware with some external device and afterwards send it to the proxy. An overview of the architecture is depicted in Figure 3.

In the above architecture, we have different networks of sensors, with each one of them being connected to a proxy, which is also a DLT node (light node). Proxies are part of a broader network of DLT validators which will include the measures values into blockchain according to the policies. Any operator could connect on a non-privileged machine to check the overall health of the system, but only the administrators could agree on changing the rules on which the system works. The gateway is located between the sensors network and the internal network with the responsibility to broadcast the incoming policies further to DLT nodes (proxies included also here). The gateway holds a record of the DLT and could be queried by other edge services.

## 4. Experimental Results

The first experimental part is focused on the distributed digital signing of policies to check the feasibility of executions times. It should be validated if a threshold approach in rules administration can match a real use case scenario.

The SMPC signing flow from this architecture can be seen as a disconnected phase since the policy are first being signed by the administrators; afterwards only on administrative changes the distributed ECDSA signing process is triggered. We measured the time for each operation in a two-party implementation, where each party resides on a different machine and communicates on Transmission Control Protocol (TCP) with the second party, the times can be observed in Table 1. These experiments were conducted on normal laptops having an Intel Central Processing Unit (CPU) (i7-4710HQ, four cores, 2.5 GHz, and 8 GB RAM) using only one CPU core and no multithreading. We choose ordinary laptops for this specific experiment, more exactly the SMPC signing flow, because the involved operations are present only in the administrative tasks, the rest of sensors and DLT nodes being simulated with limited memory or CPU time.

As it can be seen in the table above, the initial setup (key generation and backup operations) takes approximatively 5 s which is negligible since this is one time operation. The ECDSA signing, using secp256k1 curve, is the longest operation due to the communication involved between the parties during the computation, which is almost 97 kilobytes of data. The verification of a signature does not imply any collaboration between the parties.

For an extended view of execution times in larger setups of parties (multi party setup), we can look to the paper [45], where authors ran multiple experiments (from 2 to 20 parties) of distributed ECDSA signing based on a different protocol as in the [41], which we choose for our experiments.

The second part of experiments relates to the distributed ledger with the clear purpose to validate if such an approach can cope with the throughput of large networks of sensors and also to adjust the parameters in order to meet the requirements of real time processing.

Regarding the deployment of the Tendermint nodes and consensus engines, we used Docker containers for both of them. Also, for the simulation of sensors we installed a network of container instances parametrized by the number of threads. The metrics related to DLT nodes and the overall architecture with its associated parameters can be observed in Table 2.

Containers were orchestrated by Docker Swarm on two servers: Swarm manager with 128 GB RAM, 2xIntel Xeon E5-2620, 16 cores at 2.10 GHz and another swarm worker with 64GB RAM, 2xIntel Xeon E5-2603, 12 cores at 1.7 GHz. Each sensor is limited to 0.01% from CPU core and samples new random values at every 60 s. However, we did not deploy more than 1000 containers per machine because of a limitation in the Linux kernel regarding the number of ports for networking bridge on a maximum of 10 bits (this cannot be extended because of spanning tree requirements).

Based on the conducted experiments it is clear that we must increase the number of validators if we have a larger network of sensors in order to validate them faster. For example, if we take a look at the first setup, consisting of 500 sensors and five validators, we have a rate of 97.07% of transactions included in the block within the same minute of sampling compared to the setup number 3 where the rate is 98.67%. Included transactions rate is computed as the number of processed data from sensors in a sampling window (e.g., one minute) divided to the actual number of sampled data in same window (e.g., in setup number 1 DLT which would process approximately 485 values from a total of 500 sampled in a 1 min window). This requirement is not only applicable for speed optimization when validating transactions, but also implies a stronger security since Tendermint agreement might be violated in the case where more than one third of the voting power belongs to faulty validators. The block dimension increases as we have more sensors transmitting sampled data at the same time. However, the maximum block size can be modified in the Tendermint configuration file if there is such a need.

A comparison with other related works, in terms of communications, costs and key generation is not possible, since we do not add some features to the existing IoT/IIoT sensors like the PUF based authentication protocols. We outline that there are some processing delays in our proposed framework, which can be observed in Table 2 (e.g., transaction time represents the medium delay of a sampled data until it is authenticated).

## 5. Conclusions and Future Work

This research paper argues, based on the unpleasant cyberattacks and flaws in some systems, the need for a new approach to protect obsolete IIoT devices. Our proposal is based on two distributed technologies, DLT and SMPC.

Distributed Ledger Technologies bring a new decentralized paradigm that can definitely enhance the security of the Industry 4.0. As we have seen in related work, public blockchains are not suitable since the majority of them are based on a proof-of-work consensus with a very limited number of transactions that the network can handle. Other distributed registries are proposed to overcome previously mentioned issues, but none of them addresses the obsolete IIoT sensors which cannot be updated or modified.

In this paper, we presented a decentralized authentication and integrity assurance framework for IIoT devices using a private blockchain and master nodes supervising the policies pinned by the administrators. The use of DLT helps our proposed solution to be auditable and transparent, besides the distributed consensus to classify a measured value or firmware hash as authentic or not. The aim is to plug-and-play a security layer over the existing IIoT architecture, thus detecting cyber-attacks and malicious sensors in a decentralized way.

Since the ECDSA signing of rules occurs infrequently, we can argue that from this point of view the practicability of the threshold signing of policies can be achieved even in the strict operating conditions. The most frequently used cryptographic component is the validation of signatures which could be done in 0.2 ms [46] even on embedded systems with low computational resources. From this point of view we can claim that even if we are talking about smart home applications or mission-critical applications from industry we can deploy such a distributed architecture for administrator roles.

The deployed private blockchain managed to obtain an average of 97.75% of transactions per block from the total number of registered values in the sampling interval. In this regard, we can argue that the proposed DLT can efficiently handle the network traffic generated from all sensors even in a small setup (e.g., 20 nodes). By increasing the number of nodes not only that we will have a better security, but we will also have a higher rate of current transactions included within the present block.

The new proposed architecture is fully decentralized and any other centralized components were removed in order to eliminate high value single points of failure from being attacked. Thus, our proposal helps making an effective trustworthy flow of events from the moment of defining the initial policies to the moment where the operator is being alerted when some devices might act maliciously or have gone rogue.

In the future, we intend to extend the DLT implementation to smart contracts in the validation of policies for a more dynamic approach of policy control, more exactly to deploy a machine learning model in smart contracts in order to evaluate sampled data. Since ECDSA is known not to be quantum safe, we plan to implement a signing algorithm from National Institute of Standards and Technology (NIST) candidate on post-quantum standardization efforts.

## Figures and Tables

**Table 1:** This is a table of Secure Multi-Party Computation (SMPC) two-party implementation execution times.

Operation	Time
Key generation	2640 ms
Backup key	2468 ms
Signing	6464 ms
Verify	2 ms

**Table 2:** Distributed Ledger Technology (DLT) metrics and the associated parameters.

Setup No./Metric	No. of Sensors	Validator Nodes	Transaction Time	Block Time	Block Dimension ^1^
1	500	5	120.06 ms	2101.06 ms	17
2	1000	5	61.03 ms	2454.64 ms	40
3	1500	10	40.16 ms	2269.72 ms	56
4	2000	20	30.57 ms	3817.22 ms	124
5	4000	20	15.54 ms	3980.36 ms	256

^1^ Anaverage of transactions present in a block.

## References

[1] U.S. Food & Drugs Administration Firmware Update to Address Cybersecurity Vulnerabilities Identified in Abbott’s (formerly St. Jude Medical’s) Implantable Cardiac Pacemakers: FDA Safety Communication. https://www.fda.gov/medical-devices/safety-communications/firmware-update-address-cybersecurity-vulnerabilities-identified-abbotts-formerly-st-jude-medicals.

[2] Miller C., Valasek C. (2015). Remote Exploitation of an Unaltered Passenger Vehicle.

[3] Official Journal of the European Union: Regulation 679/2016 of the European Parliament and of the Council of 27 April 2016, GDPR Regulation. https://eur-lex.europa.eu/legal-content/EN/TXT/HTML/?uri=CELEX:32016R0679&from=EN.

[4] Bloomberg: The Big Hack: How China Used a Tiny Chip to Infiltrate U.S. Companies. https://www.bloomberg.com/news/features/2018-10-04/the-big-hack-how-china-used-a-tiny-chip-to-infiltrate-america-s-top-companies.

[5] Nakamoto S. Bitcoin: A Peer-to-Peer Electronic Cash System. https://bitcoin.org/bitcoin.pd.

[6] Merkle R.C. (1987). A Digital Signature Based on a Conventional Encryption Function.

[7] Merkle R.C. (1982). Method of Providing Digital Signatures. U.S. Patent.

[8] Yao A. (1982). Protocols for secure computations. FOCS.

[9] Goldreich O., Micali S., Wigderson A. (1987). How to Play Any Mental Game or a Completeness Theorem for Protocols with Honest Majority.

[10] Rabin M.O. (1981). How to Exchange Secrets with Oblivious Transfer.

[11] Even S., Goldreich O., Lempel A. (1985). A randomized protocol for signing contracts. ACM.

[12] Ishai Y., Kushilevitz E. Private simultaneous messages protocols with applications. Proceedings of the Fifth Israel Symposium on the Theory of Computing Systems.

[13] Chor B., Kushilevits E., Goldreich O., Sudan M. (1998). Private information retrieval. ACM.

[14] Shamir A. (1979). How to share a secret. ACM.

[15] Raza S., Abderlraheem M.A., Sedrati A. (2017). Blockchain and IoT: Mind the Gap. SaSeIoT.

[16] IOTA Foundation. https://www.iota.org/research/academic-papers.

[17] KSI Blockhain. https://github.com/guardtime/ksi-tool.

[18] Reyna A., Martin C., Chen J., Soler E., Diaz M. (2018). On blockchain and its integration with IoT. Challenges and opportunities. FGCS.

[19] Hyperledger Fabric. https://github.com/hyperledger/fabric.

[20] Zyskind G., Nathan O., Pentland A. (2015). Enigma: Decentralized computation platform with guaranteed privacy. arXiv.

[21] Chifor B.C., Bica I., Patriciu V.V., Pop F. (2018). A security authorization scheme for smart home Internet of Things devices. FGCS.

[22] Maltiz M., Smarzly S., Kinkelin H., Carle G. A management framework for secure multiparty computation in dynamic environments. Proceedings of the 2018 IEEE/IFIP Network Operations and Management Symposium.

[23] Košťál K., Helebrandt P., Belluš M., Ries M., Kotuliak I. (2019). Management and monitoring of IoT devices using blockchain. Sensors.

[24] Pacheco J., Hariri S. (2016). IoT Security Framework for Smart Cyber Infrastructures. FAS*W.

[25] Lin H., Bergmann N.W. (2016). IoT Privacy and security challenges for smart home environments. Information.

[26] Collen A., Nijdam A., Augusto-Gonzalez J., Katsikas S.K., Giannoutakis K.M., Spathoulas G., Gelenbe E., Votis K., Tzovaras D., Ghavami N. (2018). GHOST—Safe-Guarding Home IoT environments with personalised real-time risk control. Euro CYBERSEC.

[27] Junges P.-M., Francois J., Festor O. Passive inference of user actions through IoT gateway encrypted traffic analysis. Proceedings of the 2019 IFIP/IEEE Symposium on Integrated Network and Service Management (IM).

[28] Evrard L., Francois J., Colin J.-N. Attacker behavior-based metric for security monitoring applied to darknet analysis. Proceedings of the 2019 IFIP/IEEE Symposium on Integrated Network and Service Management (IM).

[29] Deep G., Mohana R., Nayyar A., Sanjeevikumar P., Hossain E. (2019). Authentication protocol for cloud databases using blockchain mechanism. Sensors.

[30] Hang L., Kim D.-H. (2019). Design and implementation of an integrated iot blockchain platform for sensing data integrity. Sensors.

[31] Braeken A. (2018). PUF based authentication protocol for IoT. Symmetry.

[32] Arslan S.S., Jurdak R., Jelitto J., Krishnamachari B. (2020). Advancements in distributed ledger technology for Internet of Things. Internet Things.

[33] Prada-Delgado M.A., Baturone I., Dittmann G., Jelitto J., Kind A. (2020). PUF-derived IoT identities in a zero-knowledge protocol for blockchain. Internet Things.

[34] Cacciagrano D.R., Culmone R. (2020). IRON. Reliable domain specific language for programming IoT devices. Internet Things.

[35] Shahid F., Khan A., Jeon G. (2020). Post-quantum distributed ledger for internet of things. Comput. Electr. Eng..

[36] Wang Q., Zhu X., Ni Y., Gu L., Zhu H. (2019). Blockchain for the IoT and industrial IoT: A review. Internet Things.

[37] Atlam H.F., Wills G.B. (2019). Intersections between IoT and distributed ledger. Adv. Comput..

[38] ETSI: Cyber Security for Consumer Internet of Things. https://www.etsi.org/deliver/etsi_ts/103600_103699/103645/01.01.01_60/ts_103645v010101p.pdf.

[39] OMG: Data Distribution Service. https://www.dds-foundation.org/what-is-dds-3/.

[40] Tendermint Core. https://github.com/tendermint.

[41] Unbound Tech: Unbound Blockhain-Crypto-MPC Whitepaper. https://github.com/unbound-tech/blockchain-crypto-mpc/blob/master/docs/Unbound_Cryptocurrency_Wallet_Library_White_Paper.md.

[42] SCALE-MAMBA MPC System. https://github.com/KULeuven-COSIC/SCALE-MAMBA.

[43] MPyC–Secure Multiparty Computation in Python. https://github.com/lschoe/mpyc.

[44] Sharemind. https://github.com/sharemind-sdk.

[45] Lindell Y., Nof A., Ranelluci S. (2018). Fast secure multiparty ecdsa with practical distributed key generation and applications to cryptocurrency custody. ACM CCS.

[46] Imem A.A. (2015). Comparison and evaluation of digital signature schemes employed in NDN network. IJESA.

